# The effect of PINK1/Parkin pathway on glucose homeostasis imbalance induced by tacrolimus in mouse livers

**DOI:** 10.1016/j.heliyon.2023.e15536

**Published:** 2023-04-15

**Authors:** Zhiwei Li, Jie Xiang, Shengmin Mei, Yue Wu, Yuan Xu

**Affiliations:** a Division of Hepatobiliary and Pancreatic Surgery, Department of Surgery, The First Affiliated Hospital, Zhejiang University School of Medicine, Hangzhou, Zhejiang, China; b Department of Orthopedics, Zhejiang Hospital, Hangzhou, Zhejiang, China

**Keywords:** New-onset diabetes after transplantation, Tacrolimus, PINK1, Mitochondria

## Abstract

Treatment using the immunosuppressive drug tacrolimus (TAC) is related to new-onset diabetes after transplantation (NODAT). Previous studies focused mainly on islet β cells in the diabetogenic effect of TAC. Herein, we revealed that NODAT was probably induced by TAC via hepatic insulin resistance. After daily injection of mice with TAC, a glucose metabolism disorder was induced. In addition, TAC decreased the mRNA and protein levels of insulin receptor substrate 2 (IRS2), glucose transporter type 2 (GLUT2), and the phosphorylation of protein kinase B beta (pAKT2), which indicated impaired hepatic insulin signaling. Furthermore, the PTEN-induced novel kinase 1(PINK1)/Parkin pathway was shown to have a key role in the TAC-induced imbalance of hepatic glucose homeostasis. Mechanistic investigations in human hepatic cell lines revealed that TAC stimulated PINK1/Parkin expression and inhibited the expression of insulin signaling related molecules (e.g., IRS2, GLUT2 and pAKT2). Knockdown of hepatic *PINK1* regulated downstream molecules of the PINK1/Parkin pathway (GLUT2 and IRS2), which reversed TAC-induced insulin resistance. Thus, in the liver, PINK1/Parkin signaling plays an important role in the TAC-induced imbalance of glucose homeostasis. TAC-induced diabetes might be prevented using Targeted treatment.

## 1 Introduction

Patients receiving organ transplantation commonly suffer from a severe complication termed new-onset diabetes after transplantation (NODAT) during long-term follow up [1], which is associated with a significantly increased risk of morbidity and mortality [2]. Among the many contributing factors for NODAT, immunosuppressive agents, particularly calcineurin-inhibitors (CNIs), are the main ones [3]. Tacrolimus (TAC), as the most commonly used CNI, has been considered the main contributor to NODAT [4].

Previous studies mainly focused on the β cells of islets in the diabetogenic effect of TAC [5,6]. However, a recent study found that TAC impacts both insulin secretion and insulin resistance [7]. Mitochondria are the essential organelles responsible for energy metabolism. Early study indicated that mitochondrial dysfunction was associated with the occurrence of insulin resistance [8]. The mitochondrial serine/threonine-protein kinase, PTEN-induced novel kinase 1 (PINK1), is located mainly on the outer membrane of mitochondria, and participates the regulation of mitochondrial function. The PINK1/Parkin (PRKN) signaling pathway mediates mitochondrial autophagy, named mitophagy, allowing clearance of damaged mitochondria and restoring cellular function [9]. PINK1 expression increased in the muscle tissue of obese patients with diabetes [10]. Other studies using rodent models also found that the PINK1 mRNA and protein expression levels in adipose tissue were higher in diabetic mice [11]. The liver is an important metabolic organ, exerting a vital function in the maintenance of glucose homeostasis. However, the effects of the PINK1/Parkin pathway on the liver glucose homeostasis imbalance induced by TAC remain unclear. Therefore, this study aimed to assess the influence of the PINK1/Parkin pathway on the TAC-induced imbalance of glucose homeostasis in the liver.

## 2 Materials and methods

### 2.1 Animal care and procedures

This study used C57BL/6 mice (male; 8 weeks old, weighing 20–25 g), which were supplied by the Shanghai Animal Center, Chinese Academy of Science (Shanghai, PR China). The animals were raised at 20 °C under a strict 12:12 light-dark cycle in specific pathogen-free conditions. The mice had *ad libitum* access to water and standard rodent chow (Zhejiang Academy of Medical Sciences, Hangzhou, PR China). Each mouse was kept in a plastic box, termed, an Individually Ventilated Cage, as reported previously [12]. Thirty-two mice were randomly divided into four groups, which were injected intraperitoneally daily in the morning with 0.9% saline solution (control) or TAC (TAC low-dose, 0.5 mg/kg/d; TAC medium-dose, 1.0 mg/kg/d; or TAC high-dose, 5.0 mg/kg/d). Astellas Pharma, Inc. (Tokyo, Japan) supplied the TAC. Herein, all animal procedures were carried out in accordance with the guidelines of the Institutional Animal Care and Use Committee at the First Affiliated Hospital, Zhejiang University School of Medicine and with the ARRIVE guidelines. The Ethics Committee of the First Affiliated Hospital, Zhejiang University School of Medicine approved the animal experiments.

### 2.2 Tissue harvest and glucose metabolic assays

We recorded the food intake and body weight of the mice weekly. Following overnight fasting, we collected blood samples from the caudal vein. A FreeStyle glucometer (Abbott, Maidenhead, UK) was used to measure fasting glucose every 2 weeks. An enzyme-linked immunosorbent assay (ELISA) (Millipore, Watford, UK) was used to assess plasma insulin levels. A glucose tolerance test (GTT; performed at the 8th week) and an insulin tolerance test (ITT; performed at the 9th week) were utilized [13]. The trapezoidal method was used to calculate the area under the curve (AUC) [14]. Insulin resistance (IR) was evaluated using the homeostasis model assessment for insulin resistance (HOMA-IR) with the formula: HOMA-IR = fasting blood glucose level (mmol/L) × fasting blood insulin level (mIU/L)/22.5 [15]. An IMx analyzer (Abbott Diagnostics Laboratories, Abbott-Park, USA) was used to measure the blood trough concentration of TAC via an immunoassay) at the 10th week after the initial injection [16]. Sodium pentobarbitone anesthetic overdose was injected intraperitoneally at the 10th week to humanely euthanize the mice. Blood samples and liver tissues were immediately collected. The liver tissues were divided in two: one part was frozen immediately in liquid nitrogen and placed in a −80 °C freezer; the other part was 10% formaldehyde-fixed for 24 h, dehydrated, and paraffin-embedded.

### 2.3 Histopathology

Liver morphology was assessed using hematoxylin and eosin (H&E) staining according to previously published procedures [17]. Image-Pro plus 6.0 software (Media Cybernetics, Rockville, MD, USA) was used to analyze captured images [18].

### 2.4 Cell culture and treatment

Human hepatocyte lines, HepG2 and Huh7, were purchased from the Chinese Center for Type Culture Collection (Wuhan, PR China). The hepatocytes were grown in Dulbecco's modified Eagle's medium (DMEM) (Life Technologies, Carlsbad, CA, USA) supplemented with 10% FBS and 1.0 g/L glucose in a 5% CO_2_ humidified atmosphere at 37 °C. To knock down *PINK1* expression *in vitro*, the hepatocytes were transfected with a small interfering RNA (siRNA) targeting *PINK1* (PINK1-siRNA) or siRNA-empty as a control (Genepharma, Suzhou, PR China) employing the Lipofectamine 2000 reagent (Invitrogen, Waltham, MA, USA) following the supplier's protocol [19]. At 48 h post-transfection, we harvested the hepatocytes and then co-cultured them in the presence of TAC at different concentrations (0, 5, 10, and 20 ng/mL) for 24 h. Cell viability was detected using a Countess II FL Automated Cell Counter (Thermo Fisher Scientific, Waltham, MA, USA) following the supplier's protocol.

### 2.5 RNA extraction and quantitative real-time reverse transcription PCR (qRT-PCR)

Following a previously published method [12], the TRIzol reagent (Invitrogen) was used to isolate total RNA was from liver tissues, which was then reverse transcribed to cDNA. The cDNA was used as the template for quantitative real-time polymerase chain reaction (qPCR), which was carried out on an ABIPRISM 7500 Sequence Detection System (Applied Biosystems, Foster City, CA, USA) using a SYBR Premix Dimer Eraser kit (Takara Biotechnology, Shiga, Japan). Supplemental Table 1 provides details of the PCR primers. The delta-delta cycle threshold (ΔΔCT) method was used to calculate the relative RNA expression, which was normalized to the expression of *ACTB* (encoding β-actin) in the same cDNA sample [20].

### 2.6 Western blotting

A protein extraction kit (Beyotime, Jiangsu, PR China) was used to extract proteins from hepatic cells and liver tissue. The bicinchoninic acid method (Beyotime) was used to estimate the total protein concentration. Western blotting was then carried out according to a previously published method [17]. In this study, the antibodies used were: anti-PINK1 (catalog no. 23274-1-AP, 1:1000, Proteintech, Rosemont, IL, USA), anti-Parkin (catalog no. 14060-1-AP, 1:1000, Proteintech), anti-protein kinase B beta (AKT2) (catalog no. 17609-1-AP, 1:1000, Proteintech), anti-phospho-AKT2 (pAKT2) (Ser473) (catalog no. 28731-1-AP, 1:1000, Proteintech), anti-insulin receptor (INSR) (catalog no. 20433-1-AP, 1:1000, Proteintech), anti-glucose transporter type 2 (GLUT2) (catalog no. 20436-1-AP, 1:1000, Proteintech), anti-insulin receptor substrate 2 (IRS2) (catalog no. 20702-1-1AP, 1:1000, Proteintech), and anti-β-actin (catalog no. 66009-1-Ig, 1:1000, Proteintech). The secondary antibodies comprised goat anti-rabbit IgG (catalog no. SA00001-2, 1:1000, Proteintech) or goat anti-rat IgG (catalog no. GB23302, 1:1000, Servicebio, Wuhan, PR China) conjugated to horseradish peroxidase (HRP). Immunoreactive bands were captured and analyzed using Image J software (NIH, Bethesda, MD, USA).

### 2.7 Statistical analysis

The mean ± SD was used to express all quantitative data and Student's *t*-test or the Mann–Whitney *U* test were used to assess the differences between experimental groups and controls. All statistical analyses were carried out using GraphPad-Prism 6.0 software (GraphPad Prism Software Inc., La Jolla, CA, USA). Statistical significance was accepted for a difference with a P value less than 0.05.

## 3 Results

### 3.1 TAC attenuates glucose metabolic function

**Fig. 1:**
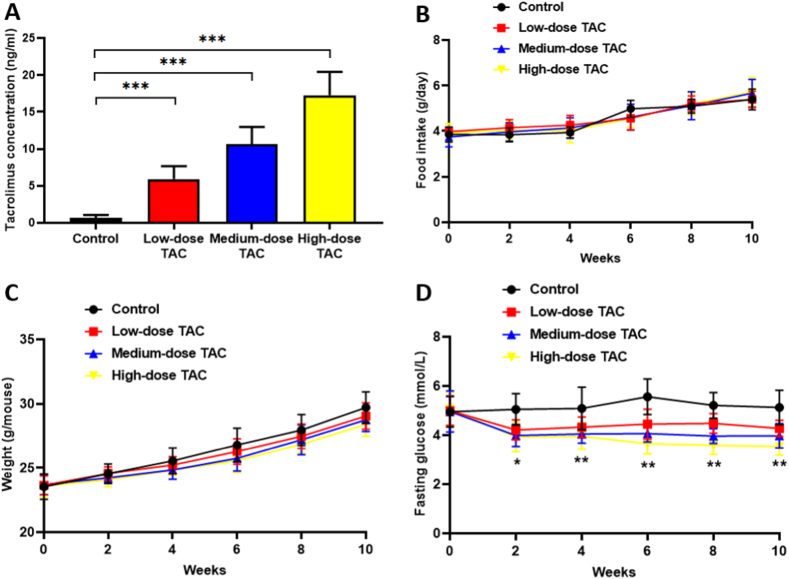
Mouse status after tacrolimus injection. (A) TAC trough concentration, (B) Food intake, (C) Body weight, (D) Fasting glucose level. *n* = 8, *P < 0.05, **P < 0.01, ***P < 0.001 in comparison with the control group. Groups: Control, saline solution; TAC low-dose (0.5 mg/kg/d); TAC medium-dose (1.0 mg/kg/d); TAC high-dose (5.0 mg/kg/d). TAC, tacrolimus.

After TAC injection daily for 10 weeks, the TAC blood trough concentrations were measured as 0.70 ± 0.36, 5.90 ± 1.78, 10.65 ± 2.30, and 17.24 ± 3.20 ng/mL in the saline control, TAC low-dose, TAC medium-dose, and TAC high-dose mouse groups, respectively (Fig. 1A). The food intake and body weight growth of the mice were not significantly different between the control group and the TAC groups (Fig. 1B–C). Interestingly, the TAC groups had significantly lower fasting glucose levels compared with that of the control group (Fig. 1D); however, the random blood sugar levels were reversed compared with those in our previous study [12].

**Fig. 2:**
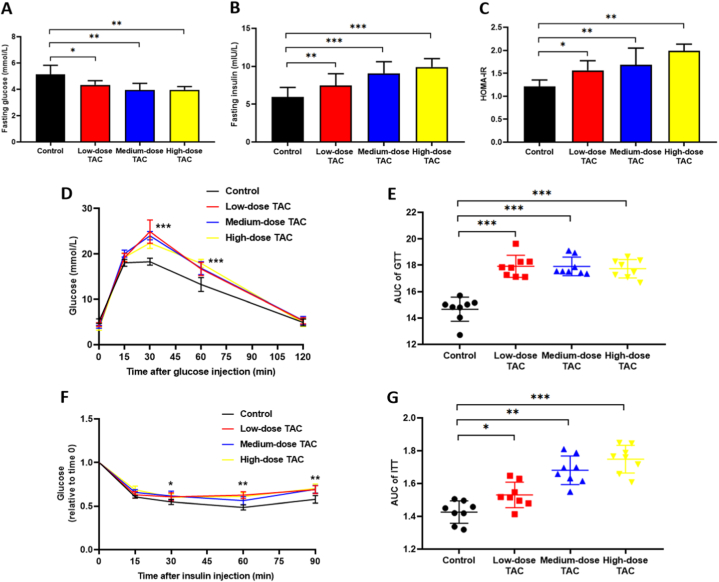
Tacrolimus led to glucose metabolic disorders. (A) Fasting glucose. (B) Fasting insulin. (C) HOMA-IR index. (D) Glucose tolerance test (GTT). (E) Area under the curve (AUC) from the GTT curve. (F) Insulin tolerance test (ITT). (G) AUC for the ITT curve. *n* = 8, *P < 0.05, **P < 0.01, ***P < 0.001 in comparison with the control group. Groups: Control, saline solution; TAC low-dose (0.5 mg/kg/d); TAC medium-dose (1.0 mg/kg/d); TAC high-dose (5.0 mg/kg/d). TAC, tacrolimus; HOMA-IR, Homeostatic Model Assessment for Insulin Resistance.

**Fig. 3:**
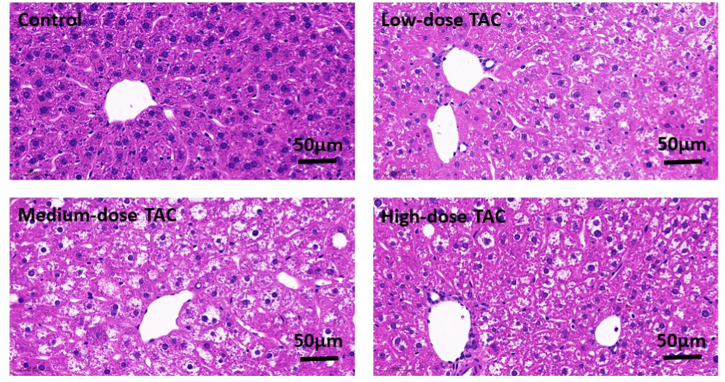
Mouse liver tissues stained with H&E ( × 400). The scale bar indicates 50 μm. Groups: Control, saline solution; TAC low-dose (0.5 mg/kg/d); TAC medium-dose (1.0 mg/kg/d); TAC high-dose (5.0 mg/kg/d).TAC, tacrolimus; H&E, hematoxylin and eosin.

Further metabolic function tests were carried out. After TAC injection daily for 10 weeks, the TAC groups showed significantly lower fasting glucose levels, and higher plasma insulin levels and HOMA-IR indexes, compared with those in the control group (Fig. 2A–C). The GTT test was then performed to evaluate changes in the physiological response to added glucose. The glucose levels in the TAC groups were significantly higher at 30 and 60 min compared with those in the control group (Fig. 2D), and the AUC of the GTT curve was significantly larger in the TAC groups (Fig. 2E). In the ITT test, following insulin injection, the glucose levels were significantly higher in the TAC groups than in the control group (Fig. 2F) and the AUC values for the ITT curves were significantly larger in the TAC groups (Fig. 2G). H&E staining showed that TAC induced fatty degeneration of hepatocytes (Fig. 3). These results indicated that TAC could induce glucose metabolic disorders, including postprandial hyperglycemia and impaired insulin sensitivity.

### 3.2 TAC causes hepatic insulin resistance

**Fig. 4:**
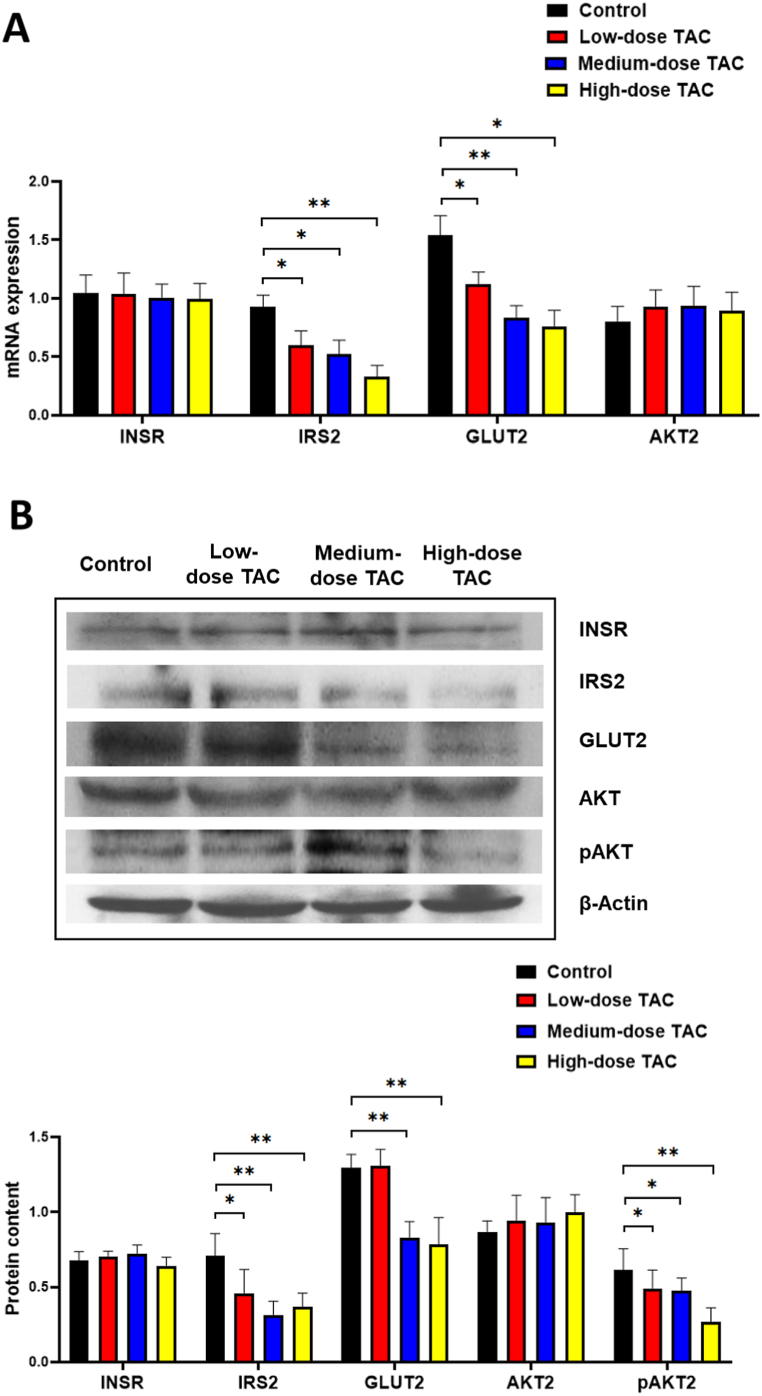
Hepatic insulin resistance was induced by TAC. (A) mRNA expression. (B) Levels of mouse proteins associated with insulin resistance in liver tissue. *n* = 8, *P < 0.05, **P < 0.01, in comparison with the control group. Groups: Control, saline solution; TAC low-dose (0.5 mg/kg/d); TAC medium-dose (1.0 mg/kg/d); TAC high-dose (5.0 mg/kg/d). TAC, tacrolimus; INSR, insulin receptor; IRS2, insulin receptor substrate 2; GLUT2, glucose transporter type 2; AKT2, protein kinase B beta; pAKT2, phosphorylated AKT2.

The results of GTT and ITT indicated that TAC caused insulin resistance. Therefore, we assessed gene transcription and protein levels in mouse liver tissues of members of the insulin signaling pathway, including INSR, IRS2, AKT2, and GLUT2. The mRNA and protein levels of IRS2 and GLUT2 were downregulated markedly after TAC treatment (Fig. 4A and B). Although there was no significant change in AKT2 expression, the level of pAKT2 decreased significantly after TAC administration (Fig. 4B). TAC seemed to have no effects on the expression of INSR in the mouse liver. These results indicated impaired hepatic insulin signaling.

### 3.3 TAC stimulates PINK1 and Parkin expression

**Fig. 5:**
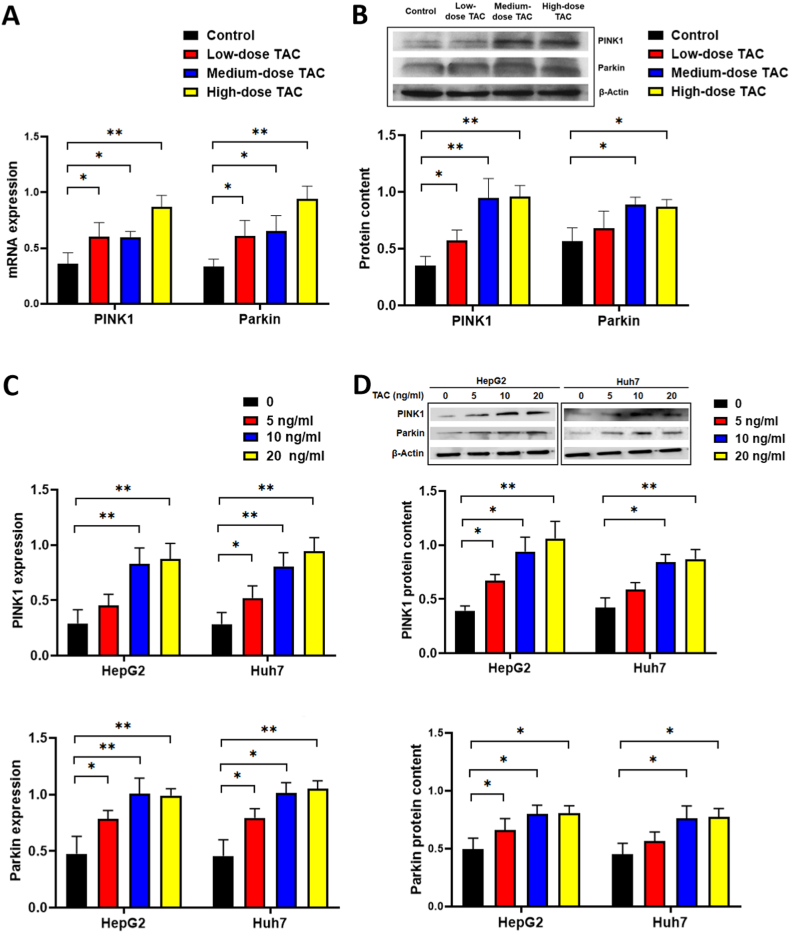
PINK1 and Parkin expression levels are stimulated by TAC. (A) *PINK1* and *PRKN* (Parkin) mRNA expression in mouse liver tissues. (B) Levels of PINK1 and Parkin in mouse liver tissues. (C) mRNA expression levels of *PINK1* and *PRKN* in human liver cells. (D) Levels PINK1 and Parkin in human liver tissues. *P < 0.05, **P < 0.01, ***P < 0.001 compared with the control group. Groups: Control, saline solution; TAC low-dose (0.5 mg/kg/d); TAC medium-dose (1.0 mg/kg/d); TAC high-dose (5.0 mg/kg/d). TAC, tacrolimus; PINK1, PTEN-induced novel kinase 1. (For interpretation of the references to colour in this figure legend, the reader is referred to the Web version of this article.)

PINK1 and Parkin are well-described regulators of mitophagy [21,22] exerting an important function in glucose metabolism [23]. Consequently, PINK1/Parkin pathway member expression was assessed in mouse livers. PINK1 and Parkin mRNA and protein levels were significantly increased in the TAC groups in comparison with those in the controls, particularly in the high-dose TAC group (Fig. 5A and B). To assess the potential mechanism of increased PINK1 and Parkin expression in TAC-treated mouse livers, we treated HepG2 and Huh7 cells with different concentrations of TAC. The results showed increased PINK1 and Parkin mRNA and protein contents in response to TAC (Fig. 5C and D). Thus, both the cellular and mouse model experiments indicated activation of the PINK1/Parkin pathway in the liver after TAC treatment.

### 3.4 TAC induced hepatic insulin resistance is associated PINK1 associated

**Fig. 6:**
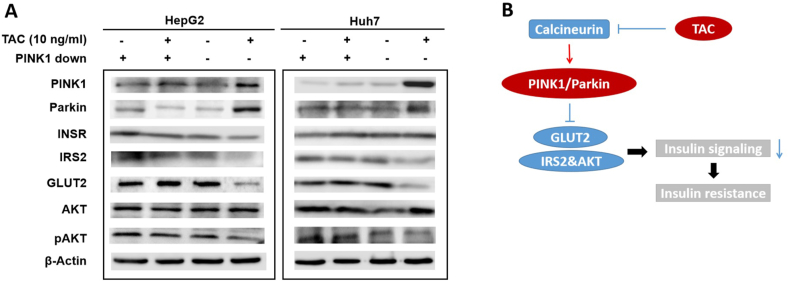
PINK1 and its downstream molecules are targeted by TAC. (A) Levels of hepatic insulin resistance-related proteins, in the presence or absence of PINK1, post-TAC administration. (B) Schematic of the hypothetical mechanism by which TAC activates PINK1/Parkin signaling, leading to downregulated expression of downstream molecules. TAC, tacrolimus; PINK1, PTEN-induced novel kinase 1; INSR, insulin receptor; IRS2, insulin receptor substrate 2; GLUT2, glucose transporter type 2; AKT2, protein kinase B beta; pAKT2, phosphorylated AKT2. (For interpretation of the references to colour in this figure legend, the reader is referred to the Web version of this article.)

Next, the influence of PINK1 on the insulin signaling pathway in hepatic cells (HepG2 and Huh7) treated with TAC was assessed by downregulating *PINK1* expression using siRNA. There were four groups for each cell line: TAC free + *PINK1* siRNA, 10 ng/mL TAC + *PINK1* siRNA, TAC free + *PINK1* siRNA-empty, 10 ng/mL TAC + *PINK1* siRNA-empty. The results indicated that knocking down *PINK1* in hepatic cells reversed the insulin resistance induced by TAC through upregulating the expression of GLUT2 and IRS2/AKT (Fig. 6A). In addition, the TAC-induced increase in Parkin expression was reversed by *PINK1* silencing in hepatic cells (Fig. 6A). Fig. 6B shows the possible mechanism by which TAC induces insulin resistance.

## 4 Discussion

As mainstay immunosuppressive agents, CNIs have been regarded as the main contributors to NODAT [5]. CNIs have a major function in dominating the balance of cellular energy metabolism [7]. The liver is a vital organ for lipid metabolism and glucose metabolism; and the liver is the main site of glucose deposition after feeding, where glucose is stored as glycogen [5]. In fact, calcineurin is widely distributed in the liver, including hepatocytes and hepatic satellite cells [24,25], therefore, the liver should be affected by CNIs, particularly TAC. Herein, the contributions of PINK1 and Parkin to TAC-induced glucose homeostasis imbalance were examined. Initially, TAC was observed to induce hepatic insulin resistance, impair IRS2/AKT signaling, and upregulate PINK1/Parkin in mice. Next, we used different concentrations of TAC in co-culture with HepG2 and Huh7 cells, respectively, which demonstrated that *in vitro* TAC treatment activated PINK1 and Parkin. Next, PINK1/Parkin signaling was suppressed to examine its effect on insulin signaling. siRNA-mediated knockdown of *PRKN* and *PINK1* abrogated the TAC-induced reduction in hepatic insulin resistance.

The present study found that long-term TAC administration could induce hypoglycemia and hyperinsulinemia in an overnight fasting state; however, hyperglycemia was in a random state. Clinically, this state was similar to that of liver transplant recipients with NODAT. Liver transplantation patients with NODAT commonly do not experience hypoglycemia; however, cases of NODAT were diagnosed based on the GTT test, not the fasting glucose test. Our GTT test results confirmed the abnormal state of glucose metabolism in mice administrated with TAC. In clinical practices, TAC shows individual variability in blood levels and has a narrow therapeutic index, so it is critical to keep optimal drug concentrations in order to avoid potential drug interactions with other drugs [26]. Our experiments in mice also showed the TAC-induced glucose metabolic disorders were dose dependent, such as fasting glucose level and fasting insulin level.

The results demonstrated that TAC could induce insulin resistance in the liver. Clinically, the HOMA-IR index is usually employed to evaluate insulin resistance, and higher HOMA-IR values were associated with NODAT [[27], [28], [29]]. Our study also observed higher HOMA-IR values in TAC-treated mice. Moreover, our results suggested that TAC-induced impairment of hepatic insulin signaling (manifested as decreased levels of GLUT2, IRS2 and pAKT) might cause the insulin resistance observed in patients with NODAT. The above results partially revealed the molecular mechanism by which TAC induced imbalanced glucose metabolism.

We also demonstrated that TAC could induce increased hepatic PINK1 and Parkin expression in mice. *In vitro* experiments further suggested that the hepatic glucose metabolic disorder induced by TAC might be mediated by PINK1. TAC stimulated PINK1/Parkin activation, resulting in PINK1 accumulation in the liver, which subsequently suppressed hepatic insulin sensitivity (via the insulin signaling pathway). Downregulation of *PINK1* in hepatic cells stimulated the insulin signaling pathway, suggesting PINK1 as a potential therapeutic target.

Recently, it has been proposed that PINK1 and Parkin have important roles in mitophagy [30], and furthermore, mitophagy is closely association with glucose metabolism [31]. However, how hepatic insulin sensitivity is affected by PINK1 and Parkin is unclear. McLelland et al. reported that PINK1 and Parkin catalyzed mitofusin 2 phosphoubiquitination, which decreased the abundance of mitofusin 2, thereby regulating the destruction of mitochondria-ER contact sites and finally suppressing mitophagy [9]. Mitophagy could eliminate oxidative stress, the accumulation of toxic lipid intermediates, and mitochondrial damage, thus counteracting insulin resistance and lipid metabolism dysfunction [32,33]. Herein, we found that TAC-induced PINK1 accumulation led to hepatic insulin resistance, which might be regulated through mitophagy-related signaling pathways. This hypothesis requires further experimental verification. According to our findings, it seems that the PINK1/Parkin pathway is related to TAC-induced hepatic insulin sensitivity impairment. Proper inhibition of PINK1 activation is of help to prevent and alleviate TAC-induced glucose metabolic disorders. These results suggest that patients with lower PINK1 storage may be more resistant to TAC toxicity and, therefore, associated with lower risk of NODAT.

This study had some limitations. First, we focused mainly on the liver. Previously, we reported TAC's effect on the gut [12], and a study of its effects on adipose and muscle tissue is in progress. Second, TAC and CsA are both classified as CNIs; however, patients treated with TAC had a higher incidence of NODAT compared with those receiving CsA [5]. The exact mechanism is unclear and more research is required to explore the different effects of TAC and CsA on glucose homeostasis in the liver. Finally, *in vitro* experiments verified that TAC-induced hepatic insulin resistance was associated with PINK1, which requires *in vivo* verification.

In conclusion, our findings showed that in mice, TAC significantly affected the glucose homeostasis of the liver via the PINK1/Parkin pathway and induced hepatic insulin resistance. Additionally, during liver graft implantation, a PINK1-targeted therapeutic strategy could help to ameliorate disorders of glucose metabolism induced by TAC.

## Author contribution statement

Zhiwei Li: Conceived and designed the experiments; Analyzed and interpreted the data; Wrote the paper.

Jie Xiang: Performed the experiments; Analyzed and interpreted the data; Wrote the paper.

Shengmin Mei: Performed the experiments.

Yue Wu: Contributed reagents, materials, analysis tools or data.

Yuan Xu, M.D.: Conceived and designed the experiments; Analyzed and interpreted the data.

## Data availability statement

Data included in article/supp. material/referenced in article.

## Declaration of interest’s statement

The authors declare no competing interests.

## Appendix A Supplementary data

Multimedia component 1 Multimedia component 1

figs1 figs1

figs2 figs2

figs3 figs3

figs4 figs4

figs5 figs5

figs6 figs6

figs7 figs7

figs8 figs8

figs9 figs9

figs10 figs10

figs11 figs11

figs12 figs12

figs13 figs13

figs14 figs14
