# Supplementary material for: The effect of PINK1/Parkin pathway on glucose homeostasis imbalance induced by tacrolimus in mouse livers
Source: Heliyon. 2023 Apr 15;9(4):e15536. doi: 10.1016/j.heliyon.2023.e15536 (PMC10161719; doi:10.1016/j.heliyon.2023.e15536)
Supplement: Multimedia component 1 [file mmc1.docx]

Table 1 Primer sequences for quantitative polymerase chain reaction

| Primer Name | Forward primer (5’-3’) | Reverse primer (5’-3’) | GenBank No. |
| --- | --- | --- | --- |
| PINK1 | TCTCAAGTCCGACAACATCCT | TTGCCACCACGCTCTACAC | NM_026880.2 |
| Parkin | CCTCTGTCATCTGGTGCCT | GCTAAGCGGTAAAGAAATCAA | NM_001317726.1 |
| INSR | AAGATGACAACGAGGAATG | GATGACAGTGGCAGGACAG | NM_001330056.1 |
| AKT2 | CCAGATGGTCGCCAACAGT | TGCCGAGGAGTTTGAGATAA | NM_001110208.2 |
| GLUT2 | AGAGGAAGTCAGGGCAAAG | GAGGATGGGCTGTCGGTAA | NM_031197.2 |
| IRS2 | AAGAGGACTTATTCCCTAACCAC | GAGGCGACCTGAACTACCA | NM_001081212.2 |
| PINK1: Phosphatase and tensin homologue (PTEN) - induced putative kinase 1; INSR: Insulin receptor; IRS2: Insulin receptor substrate2; AKT2: Protein kinase beta; GLUT2: Glucose transporter type 2. | | | |
