# Supplementary figures and images for: The effect of PINK1/Parkin pathway on glucose homeostasis imbalance induced by tacrolimus in mouse livers
Source: Heliyon. 2023 Apr 15;9(4):e15536. doi: 10.1016/j.heliyon.2023.e15536 (PMC10161719; doi:10.1016/j.heliyon.2023.e15536)

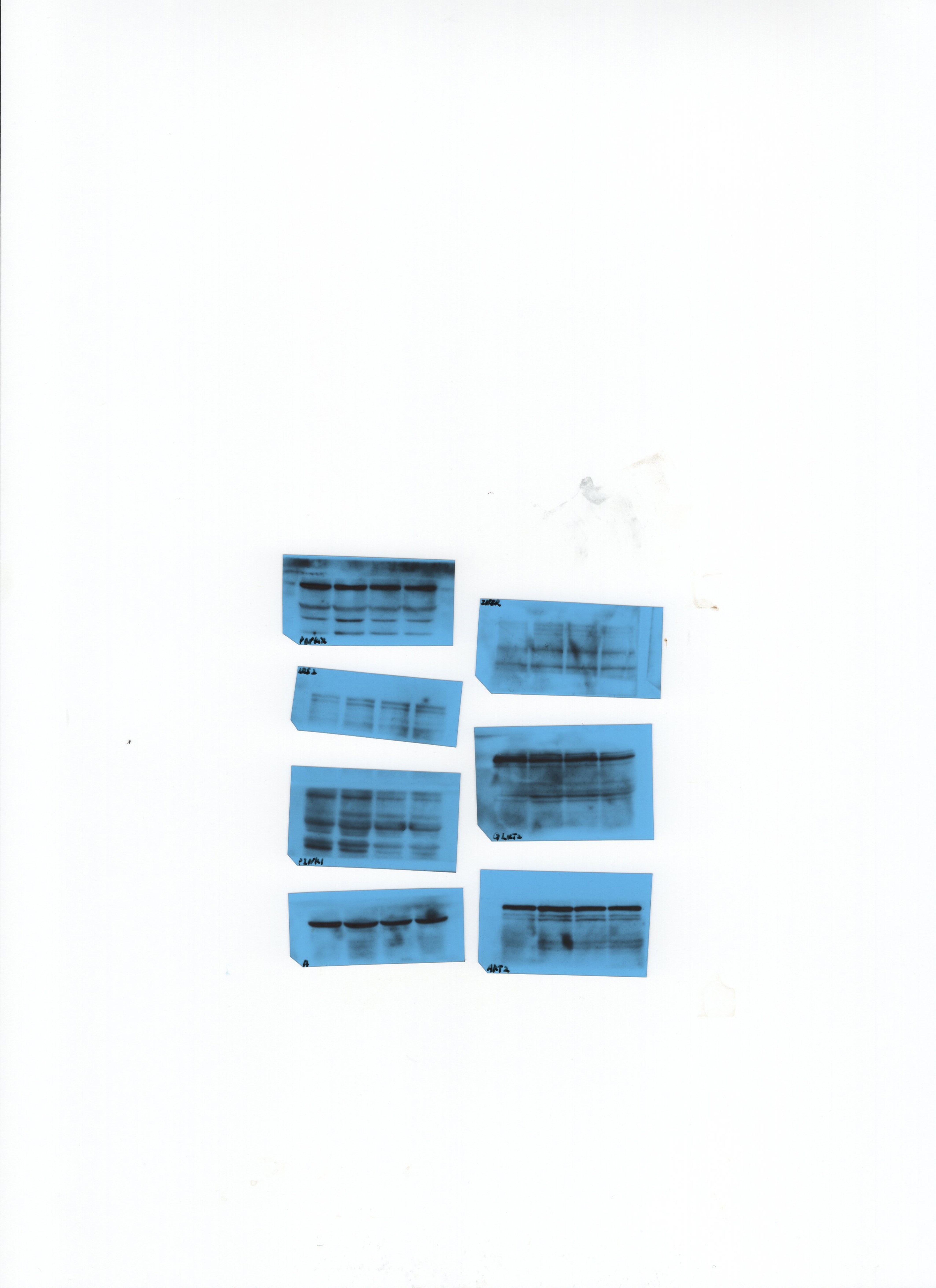

Supplement: figs1 [file mmcfigs1.jpg]

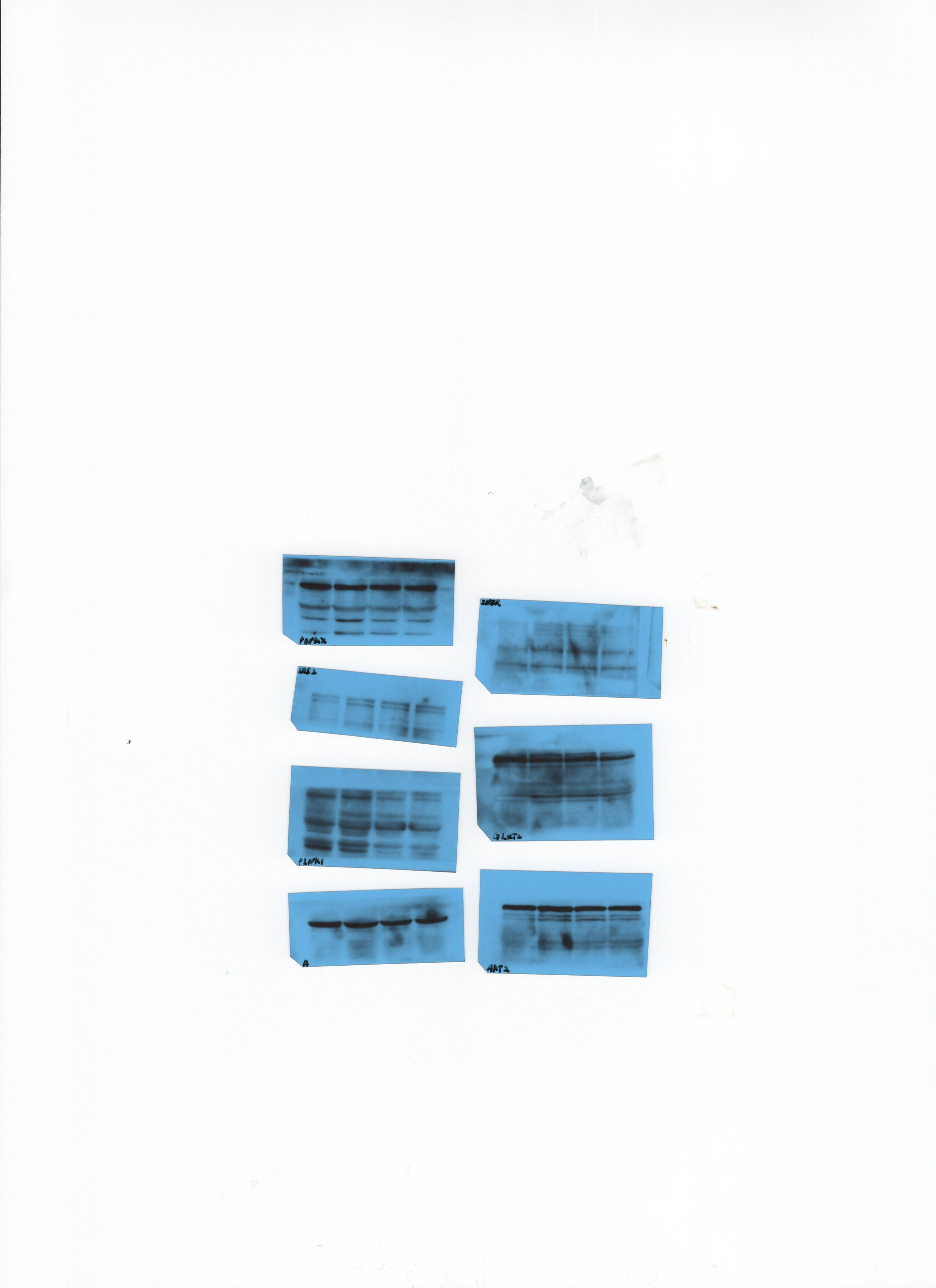

Supplement: figs2 [file mmcfigs2.jpg]

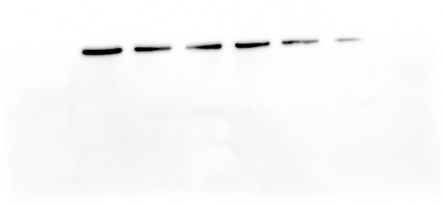

Supplement: figs3 [file mmcfigs3.jpg]

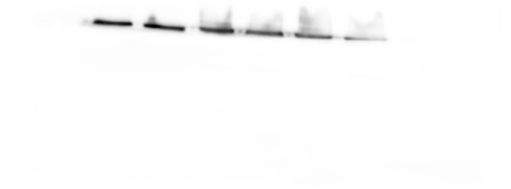

Supplement: figs4 [file mmcfigs4.jpg]

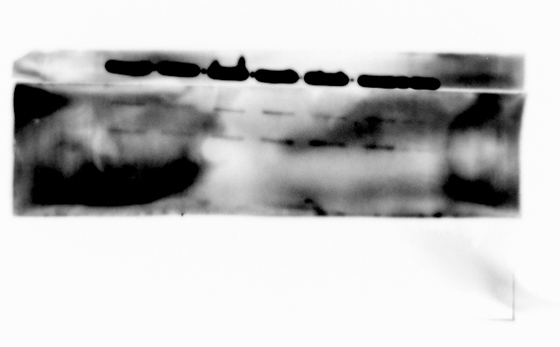

Supplement: figs5 [file mmcfigs5.jpg]

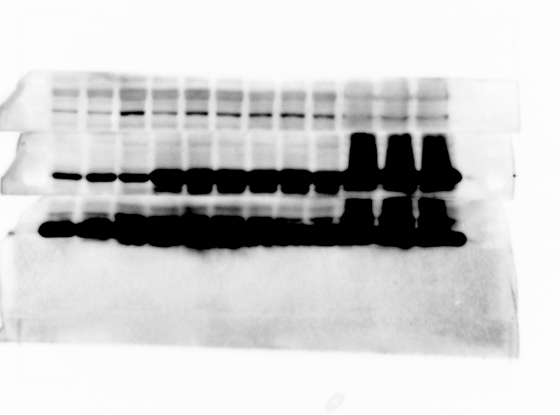

Supplement: figs6 [file mmcfigs6.jpg]

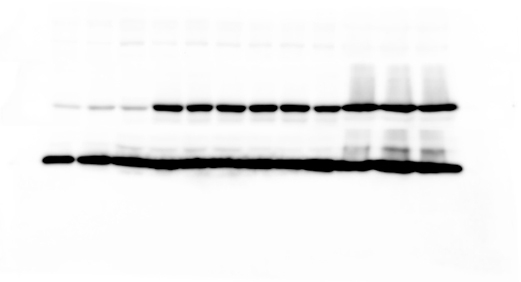

Supplement: figs7 [file mmcfigs7.jpg]

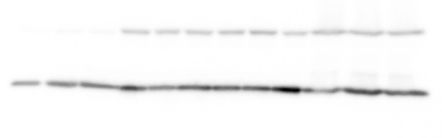

Supplement: figs8 [file mmcfigs8.jpg]

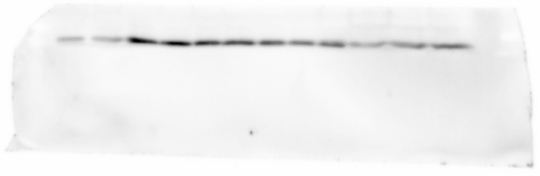

Supplement: figs9 [file mmcfigs9.jpg]

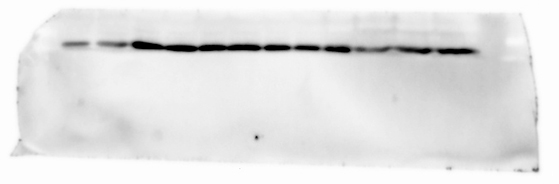

Supplement: figs10 [file mmcfigs10.jpg]

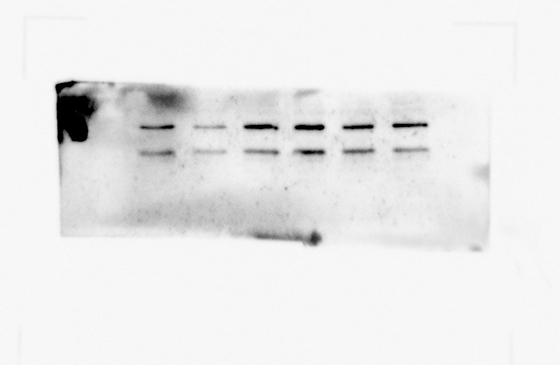

Supplement: figs11 [file mmcfigs11.jpg]

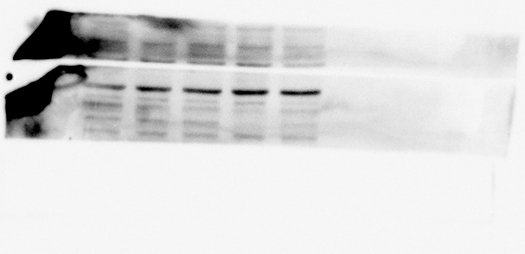

Supplement: figs12 [file mmcfigs12.jpg]

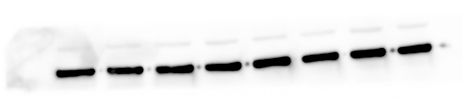

Supplement: figs13 [file mmcfigs13.jpg]

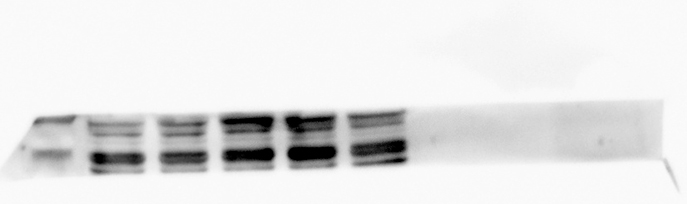

Supplement: figs14 [file mmcfigs14.jpg]
